# Electroencephalogram-Based Brain–Computer Interface and Lower-Limb Prosthesis Control: A Case Study

**DOI:** 10.3389/fneur.2017.00696

**Published:** 2017-12-15

**Authors:** Douglas P. Murphy, Ou Bai, Ashraf S. Gorgey, John Fox, William T. Lovegreen, Brian W. Burkhardt, Roozbeh Atri, Juan S. Marquez, Qi Li, Ding-Yu Fei

**Affiliations:** ^1^Hunter Holmes McGuire VA Medical Center, Department of Veterans Affairs, Richmond, VA, United States; ^2^Department of Electrical and Computer Engineering, Florida International University, Miami, FL, United States; ^3^Department of Physical Medicine and Rehabilitation, Virginia Commonwealth University, Richmond, VA, United States; ^4^Department of Biomedical Engineering, Virginia Commonwealth University, Richmond, VA, United States

**Keywords:** prosthesis, brain–computer interfaces, lower limb, control, electroencephalography, rhythm modulation

## Abstract

**Objective:**

The purpose of this study was to establish the feasibility of manipulating a prosthetic knee directly by using a brain–computer interface (BCI) system in a transfemoral amputee. Although the other forms of control could be more reliable and quick (e.g., electromyography control), the electroencephalography (EEG)-based BCI may provide amputees an alternative way to control a prosthesis directly from brain.

**Methods:**

A transfemoral amputee subject was trained to activate a knee-unlocking switch through motor imagery of the movement of his lower extremity. Surface scalp electrodes transmitted brain wave data to a software program that was keyed to activate the switch when the event-related desynchronization in EEG reached a certain threshold. After achieving more than 90% reliability for switch activation by EEG rhythm-feedback training, the subject then progressed to activating the knee-unlocking switch on a prosthesis that turned on a motor and unlocked a prosthetic knee. The project took place in the prosthetic department of a Veterans Administration medical center. The subject walked back and forth in the parallel bars and unlocked the knee for swing phase and for sitting down. The success of knee unlocking through this system was measured. Additionally, the subject filled out a questionnaire on his experiences.

**Results:**

The success of unlocking the prosthetic knee mechanism ranged from 50 to 100% in eight test segments.

**Conclusion:**

The performance of the subject supports the feasibility for BCI control of a lower extremity prosthesis using surface scalp EEG electrodes. Investigating direct brain control in different types of patients is important to promote real-world BCI applications.

## Introduction

The National Limb Loss Information Center reported that there are approximately 1.7 million people living with limb loss in the United States (1). Most of new amputations occur due to complications from impairment of the vascular system, and amputations of this type account for 82% of limb loss discharges between 1988 and 1996 (2). Lower-limb amputations account for 97% of all dysvascular limb loss discharges. A recent study of the prevalence of limb loss in the US estimated that one out of every 190 people has had an amputation, and this number may double by the year of 2050 (3).

### Advanced Lower-Limb Prosthetic Technology

People who have received limb-amputation face staggering emotional and financial lifestyle changes. They require one or more prosthetic devices and services, which must be maintained for the rest of their lives (4). A transfemoral amputee (above the knee) must expend up to 60% more metabolic energy to walk than a person with two whole legs (5) and consume as much as three times the affected-side hip power and torque (6). Commercially available prostheses comprise spring structures that store and release elastic energy throughout each walking stance period (7). Because of their passive nature, such prostheses cannot generate more mechanical energy than that is stored during each walking step. In distinction, the human ankle performs positive net work and has a greater peak power over the stance period, especially at moderate to fast walking speeds (8, 9). Emerging powered prosthetic devices with an embedded microprocessor provide a net positive power to the user, allowing more user control with less energy expenditure (4). These devices include a powered transfemoral prosthesis developed by a Vanderbilt University team led by Sup et al. (10) and a spring ankle with regenerative kinetics (SPARKy) funded by the US Army (11). In addition to the above devices for research purposes, the C-Leg (by Otto Bock, Germany) adjusts the degree and speed of knee joint swing in millisecond intervals allowing for the user to move more effortlessly. The Proprio-foot (by Ossur, Iceland) provides adaptive dorsiflexion to reduce compensation needs from the amputees in stair ambulation (though not truly, actively powered) (12). Incorporating advanced technology developed by Dr. Herr at MIT (13), *iWalk Inc*. delivers a clinically available device, BiOM^®^, a leg system [shown in the figure, adapted from Aldridge et al. (14)] that replaces combined functions of the foot, ankle, and calf regions of the human body. By adding a reflexive torque response in powered plantar flexion, the BiOM emulates the sound side stance-phase kinetics to provide better symmetry and economy of motion for amputees (15).

Prior attempts at voluntary control of the elements of a prosthesis have focused on the use of electromyographic (EMG) signals from muscle groups that remain under voluntary control. Most of this work has centered around control systems for upper extremity prostheses. Targeted muscle reinnervation is a case in point (16). This method provides adequate control but creates the extra step of muscle activation to control prosthetic functions. The Brain–Computer Interface (BCI) takes out this step for a more direct control method. Furthermore, the EMG or mechanical sensor-based control (10, 17) is reactive to the kinematic movement on residual or healthy limbs. We are striving to provide a proactive means for control that allows users to make voluntary adjustments independently before changing terrains or gait types.

Invasive BCI systems employ either spike trains or local field potentials with the brain. At the cortical surface, electrocorticography is employed. In contrast, non-invasive BCI systems employ electroencephalography (EEG) on the scalp. Invasive BCIs feature a better signal quality because electrodes are placed much closer to the neurons than non-invasive BCIs. Most of the invasive BCIs have been explored for complicated and fast control of upper extremity prosthetics (18–20). A non-invasive BCI using EEG is portable, less expensive, and provides a good time-resolution in milliseconds. However, the signal quality of the EEG may be inferior to that of signals obtained through invasive means. Non-invasive BCI can further be categorized into stimulus-induced BCIs using steady-state visual-evoked potential or SSVEP (21), the P300 evoked potential (22, 23), or combined SSVEP and P300. When using these BCI signal methods, the users need to shift their eye gaze to a visual stimulator provided by a computer monitor or LED array placed in front of them. When using stimulus-induced BCIs, the users must tolerate the strong visual flashes from the stimulators. On the other hand, the non-stimulus BCIs employ a signal method of the event-related desynchronization (ERD) and event-related synchronization (24) in EEG associated with the motor imagery, i.e., kinetic imagination of users’ limb movement without any physical movement. As this method does not require any overt motor action, it is ideal for patients with severe motor disability. For example, it can serve as a communication solution (e.g., a spelling device) for those “locked-in” persons who have totally lost motor control in conditions, such as amyotrophic lateral sclerosis patients in the late stage (25, 26).

One of the early pioneers of BCI was Dr. Jacques J. Vidal who helped establish the University of California Los Angeles Computer Science department. He coined the term “brain–computer interface” and initiated a project in that area. Since that time, studies have shown how subjects can alter images on computer screens, use a speller, reach out with robotic arms and other activities through the use of this system (27). A BCI system works through combining several systems. The initial task is to acquire a brain signal that is associated with a particular thought. This signal then is processed and amplified and funneled to drive a given device. The entire process can be compartmentalized into four phases: signal acquisition, feature extraction, feature translation, and device output. Signal acquisition can occur through surface scalp electrodes or through chips placed on or near the cortex intracranially. Prior studies have clearly demonstrated the capacity of a BCI system to control a switching mechanism. In part the results of these studies have motivated the one in this report (28).

### Unmet Needs

The control parameters of a microprocessor-driven powered prosthesis are commonly optimized for level walking. The level walking-optimized control parameters do not apply to locomotion activities other than level walking. Consequently, walking on ice or mud, for example, is not addressed with optimal parameters. Currently, amputees have extreme difficulty in going upstairs/downstairs or up/down steep-slopes when using the prosthesis optimized for level walking. This sub-optimal status leads to an increased instability and an additional load to the amputee’s intact limb (12). *How can prosthetic users efficiently adapt prosthetic parameters to altered situations and environments?* For instance, this problem exists when the user needs to go upstairs or downstairs after optimization for level walking. Amputees are frequently confronted with environmental situations that challenge their ability to ambulate efficiently and safely. Difficulty or inability in surmount such situations can significantly curtail or obstruct the restoration of a normal life. Quality of life and well-being suffer (29–31). In addition, amputees may have a higher risk of falling if the prosthesis cannot adapt to altered situations, such as stair climbing or unpaved trails (32).

Efforts have been made in state-of-the-art powered prostheses to make them more adaptive; however, the current technologies are still not sufficient. A powered prosthesis can measure user-shifted weight using biomechatronic mechanisms to adapt to the user’s weight changes; however, the microprocessor embedded in the prosthetic device has difficulty in sensing and adopting environmental changes. Because of this, the prosthesis power output is not able to adapt to the user’s needs/volitions in dynamic situations and environments. For instance, when an able-bodied person walks across a street, that person may intend to run rather than walk to reduce the risk of being hit by a car that may suddenly appear. To support the amputee’s effort to move faster, the powered prosthesis should provide increased reflex power like a biological leg. According to the biomechanical mechanism, the increased reflex power can be generated either by exerting a stronger ground reaction force or interposing a higher power gain (i.e., by parameter intervention). The former one requires that the user push harder on the residual limb to obtain an increased ground reaction force; this, however, may lead to potential damage due to the increased pressure between the socket and the residual limb (33, 34). Accordingly, the latter one is preferred as the user can receive increased power support while keeping the same level of ground reaction force. Because the prosthetic device has no ability to know the user’s desire, amputees need a new mechanism to relay their volition to appropriately affect the prosthetic control parameters so that the prosthesis can subsequently provide adaptive support. Although the other forms of control could be more reliable and quick (e.g., EMG control), the EEG-based BCI may provide amputees an alternative way to control prosthesis directly from brain.

## Purpose of This Study

**Figure 1:**
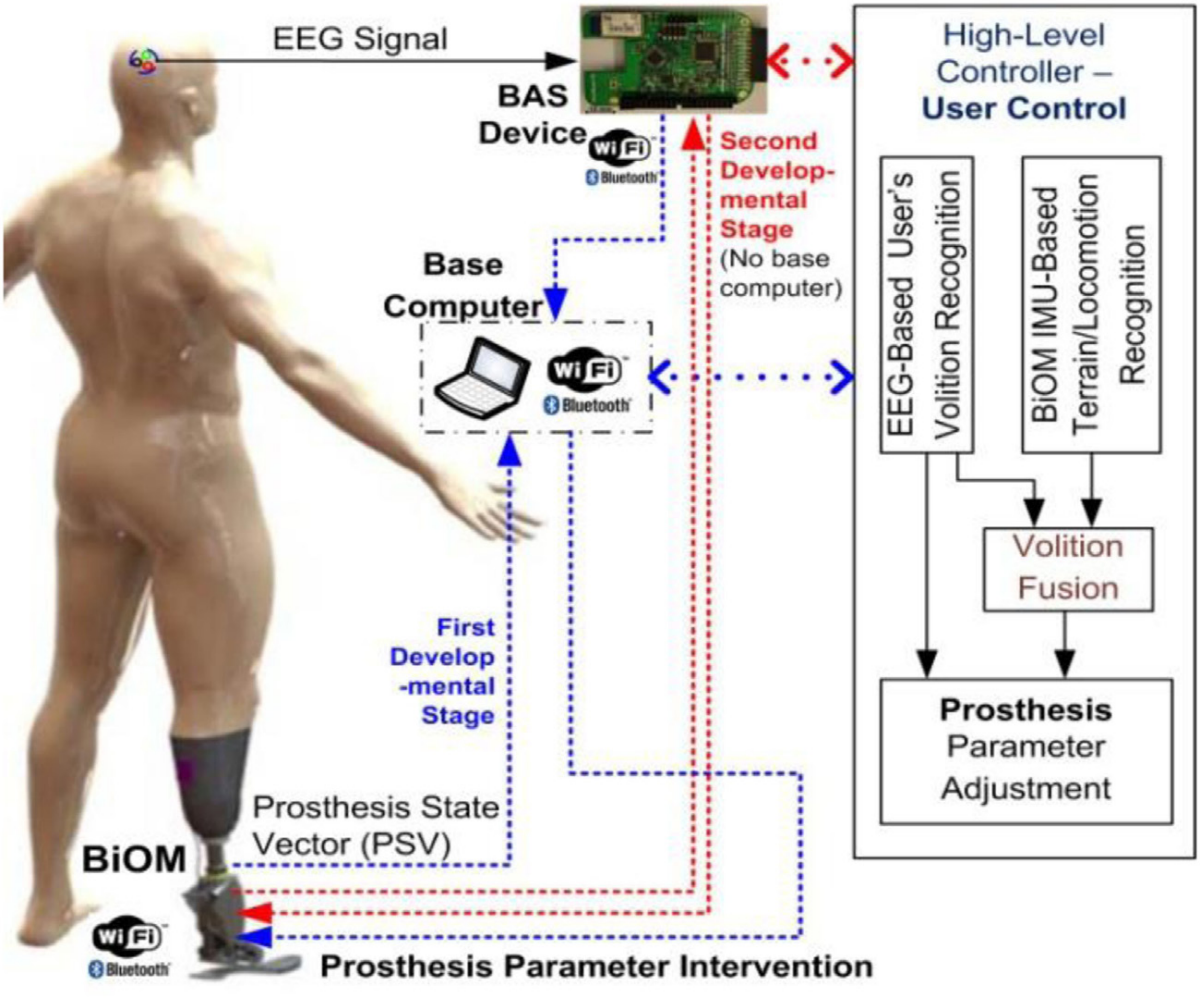
Schematic diagram of user’s direct control of prosthesis using brain–computer interface (BCI).

The prosthetic control parameters are commonly tuned to optimize level walking. User control of a prosthesis to manipulate prosthetic control parameters in real time is essential to allow for the prosthesis to adapt to altered situations and environments. Smooth, effortless user control of a prosthesis that mimics the performance of a natural biological limb can reduce the effort and the load from the user, who under the best circumstances will consume much more energy than able-bodied persons. This study proposes a volitional prosthesis control using BCIs (35, 36) to support comfortable and effortless user control of the prosthesis, in which users can control the prosthesis (parametric intervention) proactively by thought alone as shown in Figure 1. The automatic recognition of the user’s volition with subsequent automatic adjustment of prosthetic control parameters will bring amputee gait closer to normal gait patterns, which can help the amputee increase motion functions (e.g., upslope/downslope) and reduce energy expenditure in altered situations and environments. Meanwhile, recognition of the user’s conscious intent with subsequent prosthetic control will provide the user an ownership sense of “I am the one in control of prosthetic adaptation.” This sense of agency and control will improve the amputee’s psychological and physical well-being (37, 38). Although this pilot study did not achieve this end point in its entirety, it does represent an important first step toward achieving the aforementioned goals. Therefore, the first step involves providing a mechanism for the prosthetic user to adjust his prosthesis with the speed and ease of thought in response to a simple environmental circumstance. Once a single switching system has successfully been achieved, then progression can occur to multiple switching systems and other more advanced control methods that would allow the prosthetic user to respond quickly and effectively to complex environmental situations.

## Brainboard System to Support BCI-Based Prosthetic Control

Developing a powerful BCI on a platform suitable for mobile use is a challenging task that would benefit from an open platform for enabling widespread, developmental efforts. To this end, an open-source hardware solution was implemented (https://github.com/gskelly), dubbed the BrainBoard, to allow researchers and developers to easily deploy wearable EEG-based BCI systems. It will allow for wireless data transmission to a device or host computer, along with some basic onboard processing for signal enhancement and noise rejection. The board is non-specific to any electrode arrangement and allows the use of up to eight signal electrodes. The BrainBoard was designed to function as a standalone board measuring 2.1″ × 2.5 ″. In addition to the hardware design, a basic software application programming interface was developed for the BrainBoard that allowed programmers to implement BCI algorithms both standard and novel. The high-level operating capability of the BrainBoard also makes it a possible host for existing BCI software.

**Figure 2:**
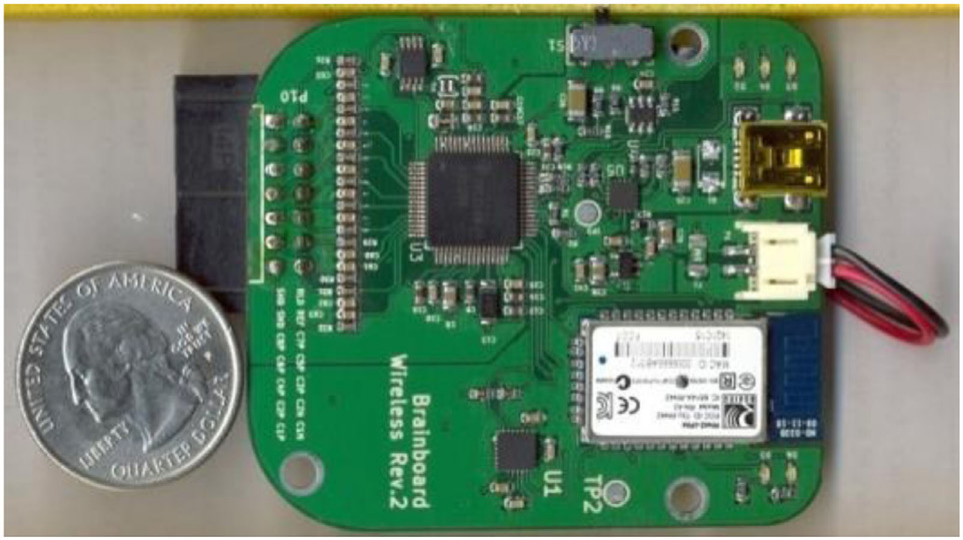
Open-source electroencephalography hardware platform.

The self-designed prototype of the light-weight, low-power consumption, battery-powered, and wireless-enabled BrainBoard for EEG/EMG recording using an ADS-1299 chip module (39) is illustrated in Figure 2. The ADS-1299 module is a low-cost, low-noise 24-bit analog front-end bio-potential measurement system recently distributed by Texas Instruments (Dallas, TX, USA). Using an ADS-1299 module greatly reduced the cost of the BrainBoard while maintaining high-quality amplification. A 32-bit MPU (AT32 Atmel AVR Microcontroller) was embedded in the BrainBoard for onboard real-time signal processing and data transmission. This self-developed BrainBoard provided high-precision EEG/EMG signal with less than 1.0 µV peak-to-peak noise. A low-power Bluetooth module RN42 (Roving Network, Los Gatos, CA, USA) was embedded to support wireless data transmission. The self-designed BrainBoard was designed to provide seamless recording and transmission of eight channels of 24-bit EEG/EMG signal with the sampling rate up to 1,000 Hz. The range of the wireless transmission can reach about 100 feet in an open space. Further, an IMU sensor was also embedded into the BrainBoard. A MPU-6050 (Gyro + Accelerometer) MEMS motion tracking chip device (InvenSense, San Jose, CA, USA) was employed. This chip provides a user-programmable gyro full-scale range from ±250, to ±2,000°/s and a user-programmable accelerometer full-scale range from ±2 to ±16 *g*, which meets the requirement for studying human locomotion.

## Case Study of BCI-Based Knee Unlock

The current study is considered a proof of concept study aiming to examine our research hypothesis on one person as a feasibility pilot work. The study provides data to optimize both hardware and software to promote the goals of BCI use in lower extremity amputees. A 36-year-old male suffered a right transfemoral amputation as a sequel of the explosion of an improvised explosive device in an overseas conflict. This person is a full time transfemoral prosthetic wearer who ambulates without any additional aids such as a cane or crutches. IRB approval of the research design was obtained, and the subject agreed to the project and signed a consent form after receiving a full explanation of the study. The subject was then trained in the use of a BCI system to activate a switching mechanism. EEG electrodes were placed on his scalp. The design of this study included visits for training and one for the actual trial with the prosthesis. The first visit trained the subject in the use of the BCI system for control of a switch on a lower extremity prosthesis. Each training visit had two sessions. In the first session, EEG recordings were made when the subject engaged in motor imagery of his limb movement. These data were utilized to determine the necessary parameters for predicting the intention to move. In the second session, those parameters were used for real-time control of the switch on the lower extremity prosthesis. After the training visits the subject then used the BCI system to control a knee-locking mechanism on the prosthesis while he walked in the parallel bars.

**Figure 3:**
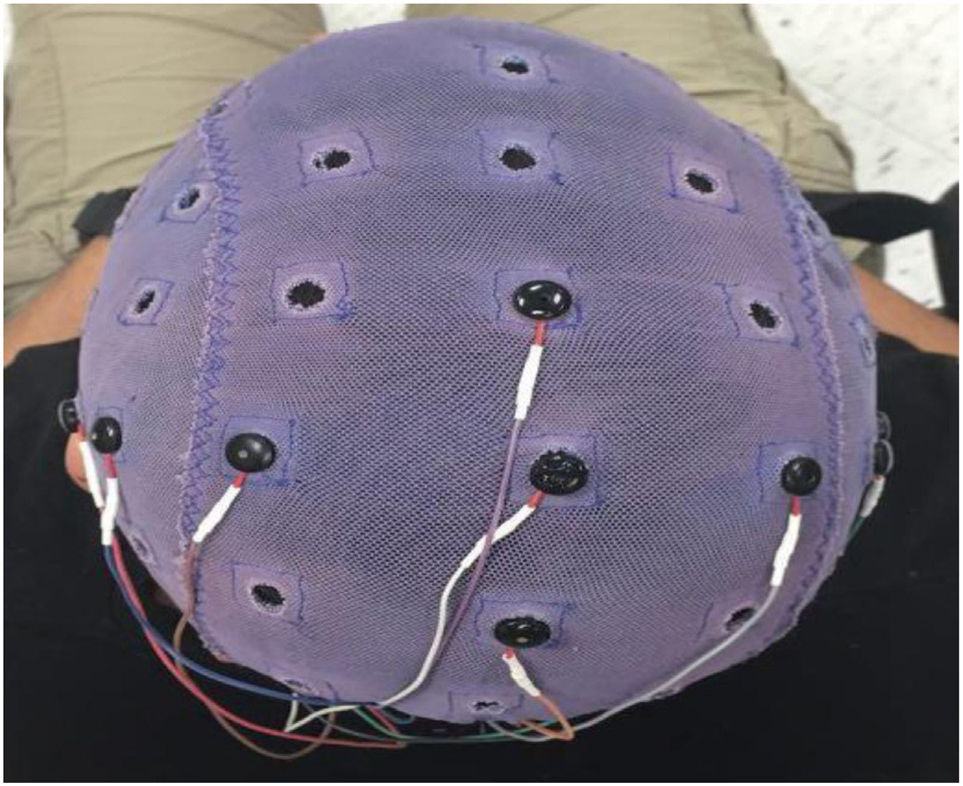
The ECI conductive electro-gel by electro-cap.com was used in this current study.

ERD was obtained from a 32 channel EEG setup with dens sampling over motor areas on both hemispheres. This method was used in our previous BCI study (36). Six Electrodes (small metal disks) were placed on the subject’s scalp over central motor areas on two hemispheres (C5, C3, C1, CZ, C2, and C4) and then secured with a plastic cap as shown in Figure 3.

**Figure 4:**
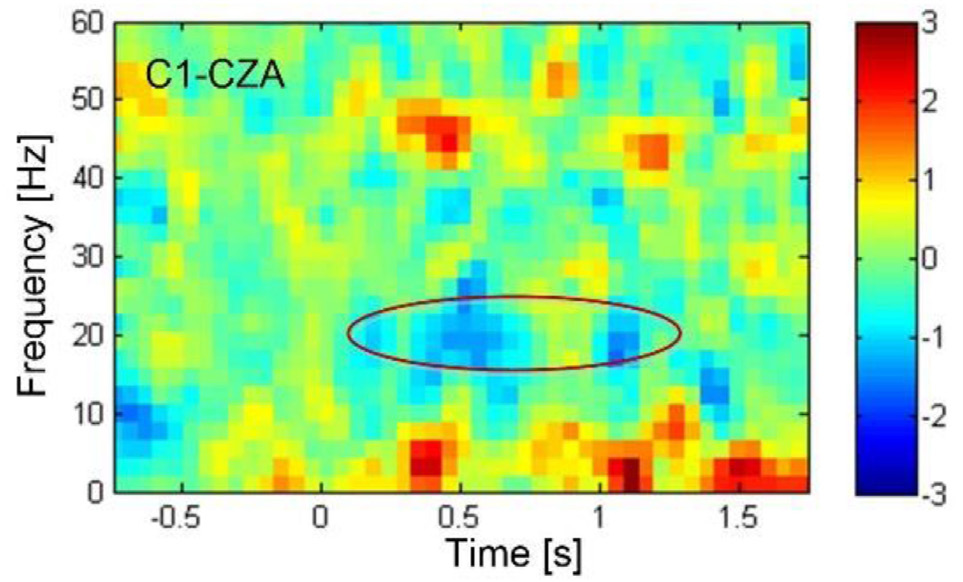
The event-related desynchronization (ERD) plotted in blue color, showing the decrease in electroencephalography rhythmic amplitude as indicated in the circle, revealed in the beta band centered around 20 Hz starting 0.5 s after the sound cue was provided at 0 s. The ERD was associated with the actual toe extension. According to the time-spectral analysis of ERD, the beta band frequency from 16 to 24 Hz were determined for feedback training and subsequently for control of knee lock.

A conductive gel was used to fill the space between the electrodes and the scalp to ensure good conductivity and minimize noise artifact. The EEG signals from the seven electrodes were referenced against the electrode on CZA (3 cm anterior to CZ). The EEG signals were amplified using a custom-made digital amplifier embedded with an ADS-1299 front-end system-on-chip bio-potential chip by Texas Instruments (Dallas, TX, USA). The EEG signals were then bandpassed (1–100 Hz) using a custom-made MATLAB tool box (BCI2VR). The ERD of the beta band (16–24 Hz) was calculated in real time against baseline activity when the subject was relaxed. The frequency band was determined by the ERD analysis of cued motor task managed in the first training session as shown in the Figure 4. An off-line linear discrimination analysis model was made for online detection of the subject’s intention to activate the switch by imaging his lower-limb movement. The algorithms as well as the BCI2VR tool box was provided in a previous study by one of the investigators (40).

Four training sessions occurred on four separate visits and were conducted before the trial. Each session lasted about 1.5 h (excluding the time for EEG setup). Custom-made software was developed to enable real time feedback from EEG. The frequency band was determined by ERD analysis. The training sessions started with wrist extension and toe extension movements on the healthy leg following a sound cue. After a reliable desynchronization in the beta band was observed, the subject was asked to imagine toe extension on the lost leg. At the beginning of the training, the ERD associated with the imagined toe extension was not reliable; the strategy was changed, and the subject was asked to move the hand, the intact leg and the residual limb. The subject was asked to imagine other types of motor activity such as walking forward. The goal was to generate a higher and more reliable ERD that could be found in the real-time feedback. In the first two training sessions, the subject was seated on a chair. The subject stood and walked during the third training session. The motor imagery task that generated the highest ERD was identified. In the fourth session, the sound cue was removed. The subject performed self-paced, imagined motor tasks, and the associated ERD was checked from the real-time feedback provided by the system.

During the training sessions, the subject was seated comfortably in an armchair with his hands and forearms supported. He was instructed to keep eye movements to a minimum including blinking, to minimize muscle action potential interference. The subject was asked to be as relaxed as possible to reduce or prevent other electrical or motion based noise. He was then asked to perform motor imagery of the amputated limb. Initially, he was asked to move the bar on a bar graph past a designated threshold point that appeared on a computer screen. When he could consistently achieve this activity more than 90% of the time, he was ready to activate the mechanism to unlock the prosthetic knee through BCI control.

A transfemoral prosthesis which is a well-fitting ischial containment socket, with gel seal-in suction liner for suspension, a modular single axis knee joint, with lock and extension assist (Otto Bock 3R33) and a solid ankle cushion heel foot, was modified with a rotary actuator that was controllable through a BCI system. The BCI program would unlock the knee, and an extension of the knee locked the prosthetic knee. The initiative to control the knee lock mechanism came from the test subject’s thought after training in the use of the BCI hardware. The rotary actuator was manufactured in the prosthetics lab through utilization of components from a myoelectric wrist rotator (Otto Bock 10S17) and was powered by a 6-V lithium ion battery. This was connected to the BCI system and the manual locking system of the knee. Once the test subject was competent and reliable in locking and unlocking the knee, this person then performed short distance ambulation in the parallel bars with one of the investigators on either side for stabilization. The test subject rose from a seated position and locked the knee. The investigators tested the success of knee locking. The subject then walked the length of the parallel bars, unlocking the knee for swing phase, turned around, returned to the wheelchair, unlocked the knee with the BCI mechanism and sat down. This procedure was repeated four times. The number of times the locking was successfully unlocked on the first effort was compared to the number of occasions in which the knee had to be unlocked. The custom-made program performed bandpass filtering and calculated ERD according to the baseline activity, which was collected when the subject was relaxing, in real time. The single-trial ERD calculated was feedback to the subject in real time without trial back-averaging. The knee lock was open only when the ERD was over a pre-set threshold. Since it was self-paced design, there was no inter-trial break. The BCI was turned off between testing segments to let the subject have a 5- to 15-min rest.

After a rest of at least 5–15 min the subject returned to the parallel bars. He came to a standing position. He walked the length of the parallel bars and back. The knee was locked for stance phase and unlocked for the swing phase. The subject took two more trips from one end to the other and back in the parallel bars. The success of unlocking the knee was again compared to occasions when unlocking was required.

After the trial the subject filled out a short survey, in which the subject indicated that the use of the BCI system was only mildly challenging to learn, and that once learned he developed complete confidence in his capacity to unlock the prosthetic knee through the system. The ultimate goal was to walk naturally. The subject was able to unlock the knee to sit in the five attempts. His success rates for the eight walking segments (each segment consisted of walking from one end of the parallel bars to the other and then a return to the starting point) were as follows: First segment, 100%; second segment, 77.8%; third segment, 100%; fourth segment, 100%; fifth segment, 50%; sixth segment, 83.3%; seventh segment, 71.4%; and eighth segment, 85.7%.

## Discussion

Human gait can be controlled either consciously or subconsciously. The EMG, including those by invasive procedures, can assist the prosthetic control without user’s conscious involvement. Under this situation, the prosthetic control is achieved subconsciously. When walking on uneven terrain or transitioning from different gait modes, the human is usually consciously involved with the locomotion control, where human preserve the “sense of control.” The proposed user’s control of prosthesis using BCI would potentially provide the user this kind of conscious control. Further, the EMG-based approach is “reactive” that the alternation in control can only be implemented after the change of locomotion mode. For example, the prosthetic control can be adapted only after walking one-stair down. In contrast, the proposed approach would potentially shift the prosthetic control before moving downstairs. Further, EMG systems are brain to nerve to muscle to electrode to device. Surface EEG electrodes are brain to device. Because there are less interfaces, there is the potential for less error and a shorter response time and less effort required on the part of the user.

Investigating direct brain control in different types of patients is important to promote real-world BCI applications. This study demonstrates that, at least on a short-term basis, non-invasive scalp recorded EEG signals can be used successfully and reliably to manipulate a lock for a mechanical knee on a prosthesis. There is debate within the literature as to whether BCI control systems can move from implanted chips attached to the brain to scalp recorded systems due to the low signal to noise (S/N) ratios in the latter. In this study the S/N ratio was improved through use of a spatial filtering system with a Laplacian array (40).

Not only did the results indicate some level of mastery of the system but the subject developed confidence in his ability without any sense of added risk. The results did show less success in the later trials. Potentially, this could indicate some level of mental fatigue. Other factors such as distractibility could have played a role. Also, the electrode conductivity could have diminished if the gel had dried or the electrodes had shifted with a degradation in the contact with the skin. In prior studies, one investigator had reduced variance in ERD through use of a longer recording window and had reduced subject fatigue through limiting the time for body action imagery to 1 s (40). Similar tactics were used in this study to enhance reliability, although there was no relaxation window. Since the actual test was managed in a real-world scenario on the self-paced mode, the accurate recording of the delay in the attempt to the knee lock operation was not available. However, the subject reported that in most cases, he could unlock the knee within a very short time.

In order for a BCI system to integrate successfully into the daily use of a prosthesis, it must perform reliably on demand and must not activate spontaneously to reduce false positives (F/P). During the trials in this study there were no observed F/P. Unintentional and unexpected unlocking of the knee during stance phase would increase the risk of falls for the user. This phenomenon was not observed. More extensive testing and training would be required to confirm this sort of reliability. This testing could also include walking in more challenging environments such as stairs or uneven terrain.

Other BCI systems for prosthetic control have focused on upper extremity control systems (41, 42). Applying the BCI technology to lower extremity prostheses potentially offers a different set of advantages. These might include prosthetic manipulation or adjustment through a hands-free mechanism, the ability to adjust rapidly according to different environmental circumstances, and a more natural appearing control of the prosthesis. Current powered and intelligent lower extremity prostheses react to the motion and demands of the user. While these can dramatically improve prosthetic function, they still only offer a strictly passive method of control that is reactive and not proactive (43). The prosthesis has no way of predicting a change in terrain or the future demands of the user. Using BCI systems the user could communicate with a prosthesis using thought alone to actively manipulate the prosthesis. This more closely approximates the natural control of a limb.

Challenges that remain for the BCI management of a lower extremity prosthesis include increasing the reliability of control and creating an adequate wireless system that is secure, dependable and wearer-friendly both for cosmesis and comfort. Furthermore, the current system only activates a switch to make a simple prosthetic adjustment. More complex systems would be desirable to increase prosthetic control options particularly for microprocessor ankle/foot systems and knees.

## Ethics Statement

All aspects of the study design was conducted based on our IRB approved protocol, all participants were given and signed an approved consent form.

## Author Contributions

DM contributed to experimental design, data collection, and writing the article. OB contributed to experimental design, data collection, data analysis, and writing the article. AG contributed to data collection and writing the article. JF, WL, BB, RA, JM, QL, and DF were involved in experimental design and data collection.

## Conflict of Interest Statement

The authors declare that the research was conducted in the absence of any commercial or financial relationships that could be construed as a potential conflict of interest.
